# Analysis of fate determination of vegetative cells with reduced phycocyanin content in a multicellular cyanobacterium using Raman microscopy

**DOI:** 10.17912/micropub.biology.000913

**Published:** 2024-02-20

**Authors:** Jun-ichi Ishihara, Yuto Imai

**Affiliations:** 1 Medical Mycology Research Center, Chiba University; 2 Faculty of International Politics and Economics, Nishogakusha University

## Abstract

The one-dimensional multicellular cyanobacterium *Anabaena* sp. PCC 7120 exhibits two different cell types under nitrogen-deprived conditions. We found that the intensity of the Raman band at 1,629 cm ^−1^ , which is associated with phycocyanin, was higher in undifferentiated cells (vegetative cells) than in differentiated cells (heterocysts). We observed cells whose band intensity at 1,629 cm ^−1^ was statistically lower than that of vegetative cells, and named them “proheterocysts”. We found that proheterocysts did not necessarily differentiate, and could divide or revert to being vegetative cells, as defined by having a higher band intensity at 1,629 cm ^−1^ .

**Figure 1. Raman band intensities at 1,629 cm -1 of individual cells at 130 hours after nitrogen depletion and transitions in cellular state at 120 and 140 hours of cells that were identified as proheterocysts at 130 hours.**
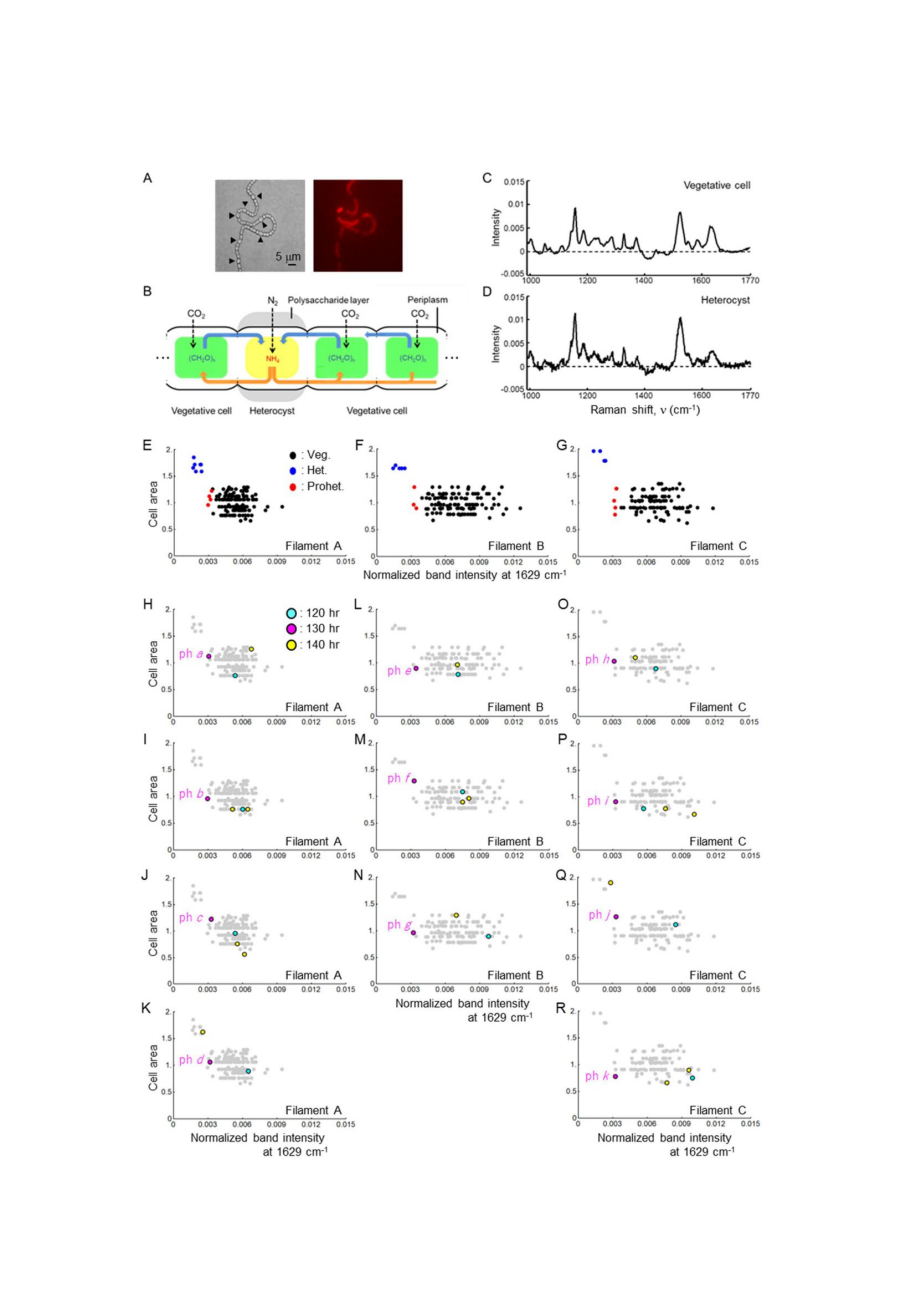
(A) Heterocyst differentiation under nitrogen source starvation conditions. Heterocysts are visible as expanded cells in which the phycobilisome complexes have been degraded. A bright field micrograph and a phycobilisome fluorescence micrograph are shown on the left and right panels. Black arrows indicate heterocysts. Scale bar = 5 μm. (B) Heterocysts and vegetative cells exchange nitrogen compounds and carbohydrates by periplasmic diffusion along the *Anabaena* filament. (C, D) An example of the normalized Raman spectra obtained from vegetative cells and heterocysts at an excitation wavelength at 785 nm. This panel is from Ishihara and Imai (2023). (E–G) Scatter plot of normalized band intensities at 1,629 cm ^−1^ and cell areas at 130 hours after nitrogen depletion. The cell area is shown as the ratio of the area of each cell in each filament to the average area of the vegetative cells in the corresponding filament. Black and blue points represent vegetative cells and heterocysts in each filament, and red points indicate proheterocysts. The number of the data points was 152, 125, and 110, which corresponds to the number of cells in Filaments A–C, respectively. Filaments A, B, and C included 7, 5, and 4 heterocysts, respectively. (H–R) Normalized band intensities at 1,629 cm ^−1^ and cell areas at 120 and 140 hours of cells identified as proheterocysts at 130 hours were superimposed on the scatter plots of the respective filaments at 130 hours (that is, Figures E–G). The gray points in each graph are the same as the black and blue points in Figures E–G. The magenta points represent data from proheterocysts identified at 130 hours (“ph” on Figure H–R stands for proheterocyst). The cyan and yellow points represent data at 120 and 140 hours from cells identified as proheterocysts at 130 hours. Thus, the cyan, magenta, and yellow points show data from the same cells at different time points after nitrogen depletion. When two yellow points are shown in a single graph (Figures I, J, M, P, and R), they represent the sister cells generated by division of a proheterocyst identified at 130 hours.

## Description

*Anabaena* sp. PCC 7120 (hereafter referred to as *Anabaena* ) is one kind of the multicellular cyanobacteria, and shows a one-dimensional morphology composed of many vegetative cells. These cells express Photosystem I (PSI) and II (PSII) to carry out photosynthesis. Under conditions free of nitrogen compounds, several vegetative cells differentiate into nitrogen-fixing heterocysts at almost 10-cell intervals (Golden et al., 2003; Kumar et al., 2009; Ishihara et al., 2015) (Figure A). Once differentiated, a heterocyst never divides or dedifferentiates. After an increase in the number of vegetative cells due to the division, heterocysts differentiate newly in an almost intermedium region between older heterocysts. Heterocysts can be easily identified with optical microscopy because their size and shape are larger and rounder than those of vegetative cells. In addition, phycobilisome complexes, which are a crucial element of PSII, chemically decompose or are not activated in heterocysts (Zhang et al., 2006) . As photosynthesis and nitrogen fixation are incompatible reactions (Meeks et al., 2002) , heterocysts and vegetative cells exchange their respective metabolites with each other (Figure B).

We previously studied microbial pigment composition in vegetative cells and heterocysts of *Anabaena* with Raman spectral measurements (Ishihara et al., 2013; Ishihara and Takahashi, 2023, Ishihara and Imai, 2023) (Figures C and D). The Raman spectral bands were assigned to vibrations of the light-harvesting pigments chlorophyll *a* , carotene, phycocyanin, and allophycocyanin at an excitation wavelength of 785 nm (Ishihara and Takahashi, 2023) . The band positions in the Raman spectra of the vegetative cells were almost identical to those of the heterocysts; however, the band intensities exhibited some distinctive difference. In particular, the intensity of the Raman band for phycocyanin was significantly lower in heterocysts than in vegetative cells (Ishihara and Takahashi, 2023; Ishihara and Imai, 2023) . Considering that phycocyanin is a component of the light-harvesting phycobilisome complex, the findings from our previous study correlate well with those from earlier studies that reported that phycobilisomes decompose during differentiation (Asai et al., 2009) . Furthermore, our study established normalized intensity at 1,629 cm ^−1^ as being representative of phycocyanin content (Ishihara and Takahashi, 2023; Ishihara and Imai, 2023) .

Vegetative cells that exhibited a statistically significant decrease in Raman band intensity at 1,629 cm ^−1^ were considered to be candidates for future heterocysts (Ishihara and Imai, 2023) . In our previous study, we named such cells “proheterocysts”; these cells have begun to lose their phycobilisome structures, yet are still functionally and morphologically vegetative cells (Ishihara and Imai, 2023) . In our previous study, we identified seven proheterocysts in three *Anabaena* filaments and confirmed that one of them finally differentiated into a heterocyst (Ishihara and Imai, 2023) . However, whether the other six proheterocysts had differentiated, until 8 hours after the Raman spectral measurement was taken, was impossible to check. Thus, the aim of the current study was to investigate how proheterocysts form and determine whether they invariably differentiate into mature heterocysts.

To address these questions, we first measured the Raman spectra of individual cells along an *Anabaena* filament at 120, 130, and 140 hours after nitrogen depletion. At 130 hours, we identified proheterocysts among the vegetative cells by their statistically lower band intensities at 1629 cm ^−1^ . We then determined the intensity value at 1,629 cm ^−1^ and the phenotype (vegetative cell or heterocyst) at 120 and 140 hours of all cells identified as proheterocysts at 130 hours. We adopted the definition of proheterocyst used in our previous study (Ishihara and Imai, 2023) : the band intensity at 1,629 cm ^−1^ of the proheterocysts was more than two standard deviations greater than the average band intensity at 1,629 cm ^−1^ of all vegetative cells in each filament.

We analyzed the Raman spectra of individual cells from three *Anabaena* filaments (Filaments A–C). The segment length, which was defined as the number of vegetative cells surrounded by two heterocysts, was 19.33±13.35, 15.50±4.65, and 21.33±8.74 (avg±s.d.) in Filaments A, B, and C, respectively, at 130 hours. The Raman spectra were obtained as we described previously (Ishihara and Takahashi, 2023; Ishihara and Imai, 2023) , and as explained in the Methods. The intensity values of the Raman spectra from 990 to 1,770 cm ^−1^ were normalized to unity. The ranges of the band intensities at 1,629 cm ^−1^ among the vegetative cells and heterocysts were 0.00295–0.0126 and 0.00134–0.00243, respectively. Especially, the upper limit of the band intensity among the heterocysts was lower than the lower limit among the vegetative cells in all filaments. That is, all vegetative cells exhibited distinctively higher phycocyanin band intensities than did heterocysts.

We first plotted the normalized band intensity at 1,629 cm ^−1^ against cell area at 130 hours to detect fluctuations in phycocyanin content in individual cells (Figures E–G). There were three, four, and three proheterocysts in Filaments A, B, and C, respectively (red dots in Figures E–G). Given that the cell areas of the proheterocysts were comparable to those of the vegetative cells, phycocyanin decomposition was considered to have already begun in the proheterocysts. Next, we superimposed the band intensities at 1,629 cm ^−1^ and the cell areas at 120 and 140 hours of cells identified as proheterocysts at 130 hours onto the scatter plots of data from their respective filaments at 130 hours (Figures H–R). As Figures H–R show, the band intensities at 1,629 cm ^−1^ of cells identified as proheterocysts at 130 hours were not all statistically lower than those of vegetative cells at 120 hours. This suggests that the cells transitioned from vegetative cells to proheterocysts during this 10-hour time period. At 140 hours, however, the proheterocysts identified at 130 hours exhibited three distinct cellular fates. First, proheterocysts *d* and *j* in Filaments A and C at 130 hours had differentiated into heterocysts by 140 hours (Figures K and Q). The new heterocysts were as large as the preexisting heterocysts, and their band intensities at 1,629 cm ^−1^ had decreased to a level comparable to those of heterocysts at 140 hours. Second, proheterocysts *b* , *c* , *f* , *i* , and *k* in Filaments A–C at 130 hours had each divided into two vegetative cells at 140 hours (Figures I, J, M, P, and R). The cell areas and band intensities at 1,629 cm ^−1^ of the newly divided cells were similar to those of the preexisting vegetative cells. Third, proheterocysts *a* , *e* , *g* , and *h* in Filaments A–C at 130 hours had not differentiated or divided by 140 hours, and exhibited a phycocyanin content similar to that of vegetative cells at 140 hours (Figures H, L, N, and O). The cell areas were increased but still within that range of vegetative cell areas, and the band intensities at 1,629 cm ^−1^ were not statistically lower at 140 hours.

In conclusion, proheterocysts do not necessarily differentiate into heterocysts. When proheterocysts do not differentiate, the proheterocysts may divide, or they may neither divide nor differentiate. When the proheterocysts divided, the Raman band intensities at 1,629 cm ^−1^ of the daughter cells were restored to levels similar to those exhibited by other vegetative cells (except for proheterocysts). However, even when the proheterocysts did not divide or differentiate, their Raman band intensities at 1,629 cm ^−1^ were restored; that is, the proheterocyst reverted to being a vegetative cell. In addition, all of the proheterocysts were generated from vegetative cells that exhibited similar Raman band intensities at 1,629 cm ^−1^ to other vegetative cells. Therefore, we propose that proheterocysts represent a transient precursor state to differentiation and may reversibly revert to the vegetative cell state.

## Methods

**Bacterial strains and culture**

*Anabaena* sp. PCC 7120 (wild type) were cultured in 25 ml of BG-11 _0_ (lacking sodium nitrate) liquid medium at 30℃ under white fluorescent light (FL30SW-B, Hitachi co.) illumination of 45 μM photons m ^-2^ s ^-1^ . The culture was shaken and incubated at 120 rpm until the optimal density at 730 nm (OD _730_ ) was 0.4–0.5. The liquid culture was washed three times with BG11 _0_ liquid medium, diluted to an OD _730_ of about 0.2 and placed under a fresh BG-11 _0_ solid medium plate containing 1.5 % agar solution (Becton, Dickinson and company, USA) with a bottom dish glass. The sample was placed in a Raman microscope (as see below) kept at 30℃ under illuminated with a white fluorescent lamp at 45 μM photons m ^-2^ s ^-1^ .

**Raman microscope and spectral pre-treatments**

We used In Via confocal Raman spectrometer equipped with a CCD camera (inVia Reflex, Renishaw co.) to measure the Raman spectrum. The excitation wavelength was at 785 nm. The central points of the cells were chosen along each filament and the Raman spectra of individual vegetative cells and heterocysts were measured. A typical Raman spectrum of a small confocal volume in the cytoplasm (horizontal diameter, ~ 1 μm) of a single living vegetative cell (~ 3 μm diameter) provides a signal-to-noise ratio sufficient for the analysis (~1 s per pixel, with a 785 nm laser at ~20 mW directed at the confocal volume). In this study, the baselines of Raman spectra were corrected. The baseline-corrected Raman spectrum *y* ’( *ν* ) was calculated as *y* ’( *ν* ) = *y* ( *ν* ) - *y*_poly_ ( *ν* ), where *y*_poly_ ( *ν* ) is a fitted polynomial curve constructed using the following procedures. (i) For a spectrum truncated between the minimum and maximum Raman shift positions *ν*_min_ and *ν*_max_ , a polynomial function was used to select the order *d* of the function that fits the baseline ( *d* =3). (ii) Using the least squares method, the polynomial function *y*_poly_ was first fitted to the Raman spectrum *y* . (iii) The Raman spectrum *y* was split up and down with respect to the fitted baseline *y*_poly_ . (iv) The number of data points above *y* was defined as *N*_A_ , and the number of data points below *y* was defined as *N*_B_ . When *N*_B_ was larger than *N*_A_ , the upper part of *y* was removed from the whole *y* , and the Raman spectrum *y* was replaced by the lower part of the spectrum. The procedure (ii) was then repeated. When *N*_A_ was larger than *N*_B_ , the baseline was considered to be the best fit and optimal.

**Measurement of the cell area**

We measured the cellular area by using Image J software (ver. 1.54f, NIH).
